# Measuring gender norms about relationships in early adolescence: Results from the global early adolescent study

**DOI:** 10.1016/j.ssmph.2018.10.014

**Published:** 2018-10-31

**Authors:** C. Moreau, M. Li, S. De Meyer, Loi Vu Manh, G. Guiella, R. Acharya, B. Bello, B. Maina, K. Mmari

**Affiliations:** a Department of Population, Family and Reproductive Health, Johns Hopkins Bloomberg School of Public Health, Baltimore, MD, United States; b Gender, Sexual and Reproductive Health, CESP Centre for Research in Epidemiology and Population Health, U1018, Inserm, F-94805 Villejuif, France; c International Centre for Reproductive Health (ICRH), Department of Uro-Gynecology, Faculty of Medicine and Health Sciences, Ghent University, Ghent, Belgium; d Institute of Sociology, Vietnam Academy of Social Sciences, Vietnam; e Institut Supérieur des Sciences de la Population, University of Ouagadougou, Burkina Faso; f Population Council, New Delhi, India; g Academy for Health Development (AHEAD), Ajanaku Estate, Ile-Ife, Nigeria; h Institute of Child Health, Faculty of Public Health, University of Ibadan, Ibadan, Nigeria; i Population Dynamics and Sexual and Reproductive Health and Rights Unit, African Population and Health Research Center, Nairobi, Kenya; j School of Public Health, University of Witswatersrand, Johannesburg, South Africa

**Keywords:** Gender norms, Measurement, Adolescence, Cross cultural research

## Abstract

**Introduction:**

Gender norms are increasingly recognized as drivers of health and wellbeing. While early adolescence constitutes a critical window of development, there is limited understanding about how adolescents perceive gender relations across different cultural settings. This study used a mixed-method approach, grounded in the voices of young people around the world, to construct and test a cross-cultural scale assessing the perceptions of gender norms regulating romantic relationships between boys and girls in early adolescence.

**Methods:**

The study draws on the Global Early Adolescent study (GEAS), a study focusing on gender norms and health related outcomes over the course of adolescence in urban poor settings worldwide. In-depth interviews were first conducted among approximately 200 adolescents between 10–14 years in seven sites across 4 continents to identify common scripts guiding romantic relations in early adolescence. These scripts were then transformed into a multidimensional scale. The scale was tested among 120 adolescents in each of 14 GEAS sites, followed by a second pilot among 75 adolescents in six sites. We evaluated the psychometric criteria of each sub-scale using principal component analysis, and parallel analysis, followed by exploratory factor analysis to guide the selection of a more parsimonious set of items.

**Results:**

Results suggested a two-factor structure, consisting of an “adolescent romantic expectations” subscale and a “Sexual Double Standard” subscale. Both subscales yielded high internal validity in each site, with polychoric Cronbach alpha values above 0.70 with the exception of Kinshasa for the adolescent romantic expectations scale (0.64) and Hanoi for the sexual double standard scale (0.61).

**Conclusion:**

This study reveals common perceptions of gendered norms about romantic engagement in early adolescence, normative for both sexes, but socially valued for boys while devaluated for girls. The findings illustrate that social hierarchies of power in romantic relationships form early in adolescence, regardless of cultural setting.

## Highlights

• Gender norms about romantic relationships prevail across diverse cultures.
• New cross-cultural scales measure early adolescents’ perceptions of gender norms;
• Adolescent romantic relations are viewed as normative but damaging for girls;
• Even before sexual activity, adolescents understand societal expectations about relationships;
• Social hierarchies of power in relationships form in early adolescence.

## Introduction

Gender norms—the collective and often unequal expectations about how women and men should behave, feel, think and interact in a given society—are increasingly recognized as a critical social driver of health and wellbeing (Doyal, 2000, Connell, 2012). Transforming patriarchal norms to promote gender equality is not only a human rights imperative but also a strategy for improving women and girls’ health and wellbeing, inscribed in sustainable development goal 5 on gender equality (United Nations Economic Council, 2017). Over the last two decades, a large body of work has demonstrated the interrelation between gender inequalities and women’s health (Doyal, 2000), through processes of discrimination, abuse and unequal access to resources. The ways gender norms inform male health, extensively described in the context of HIV acquisition among men who have sex with men, is also receiving growing attention as it pertains to male heterosexual health and wellbeing (Pleck et al., 1994, Courtenay, 2000).

The mechanisms linking gender norms to individual behaviors are the subject of intensive theoretical discussion (Mackie, 2018). According to Cialdini, gender norms, which include collective beliefs about what people do (descriptive norms) and what others think they *should* do (normative expectations) serve as scripts, for acceptable behavior based on one’s sex (Cialdini, Kallgren, & Reno, 1991). Gender scripts are enforced through mechanisms of social sanctions which are particularly effective for limiting behavior to a prescribed set of actions which align with ideas of masculinity or femininity. These scripts pertain to all spheres of life, including health behaviors, which serve as means to demonstrate manhood or womanhood (Courtenay, 2000, Connell, 2012). Gender norms are particularly salient in informing relational health outcomes, such as sexual and reproductive health. A number of studies have tied hegemonic forms of masculinities to unsafe sexual interactions, by encouraging male sexual risk taking (Barker et al., 2010, Jewkes et al., 2015), and by predisposing women to a wide range of sexual health risks (Jewkes and Morrell, 2010), including intimate partner violence (Jewkes, 2002), unprotected intercourse, STI/HIV acquisition (Krishnan et al., 2008) and unintended pregnancy (Pallitto & O’Campo, 2005). Together these risks represent the most important contributor to disability and death adolescent girls worldwide (15–19 years) (Patton, Sawyer, Ross, Viner, & Santelli, 2016).

Given the growing recognition that adolescence serves as a foundation for lifelong health trajectories (Patton et al., 2016), it is important to draw attention to the ways gender expectations regulate behaviors in this critical stage of development (Patton, Darmstadt, Petroni, & Sawyer, 2018). Indeed, while gender norms are internalized and enacted throughout the life course, research suggests an intensification in early adolescence (Hill and Lynch, 1983), marked by an expansion of young people’s ability to adapt and engage in a variety of social interactions, while still depending on the knowledge, power and resources of others. Such interactions are critical in the ways individuals co-construct their belief systems, which in turn inform their behavior and their understanding of self as emerging adults. Apart from one recent study conducted in Nepal, suggesting a benefit of intervening early among very young adolescents (Lundgren, Beckman, Chaurasiya, Subhedi, & Kerner, 2013), there is little insight about the ways gender norms manifest and solidify in early adolescence. Cross-cultural studies, such as the Global Early Adolescent Study (described in more detail below) are also needed to learn about the similarities and differences of this process across settings (Basu et al., 2017, De Meyer et al., 2017) and inform the development of cross-cultural measures. Such measures are important to track progress towards meeting SDG 5 goals and to assess the short and long-term impact of gender transformative interventions for early adolescent boys and girls globally.

One of the limiting factors for research on this topic is the lack of validated measures for assessing gender norms in early adolescence across diverse cultural settings. Current measures mostly apply to older adolescents or young adults (Emmerink, Eijnden, Bogt, & Vanwesenbeeck, 2017) and tend to focus on norms about masculinities (Pleck et al., 1994) or gender equality (comparing girls to boys or vis versa) (Pulerwitz & Barker, 2008). The widely used Gender Equitable Men (GEM) scale, initially developed for adolescents and young adults 15–24 years, focuses on gender equitable and unequitable norms as they relate to intimate relationships, gender based violence and sexual behaviors (Pulerwitz & Barker, 2008). The measure has proven internally reliable across cultures with some adaptation (Gottert et al., 2016) and has been used to predict sexual health outcomes among older populations (Stephenson, Bartel, & Rubardt, 2012). However, many of its items do not relate to the experiences of early adolescents, who have never engaged in sexual relations and may have difficulty reflecting on abstract situations. A recent study conducted in Uganda tested the psychometric properties of the GEM scale for younger adolescents suggesting 16 of the original 24 items loaded above 0.3 on a single factor (Vu et al., 2017). However, the validity of this construct in other settings has not been tested. A few measures, all developed in the United States, have specifically focused on early adolescence, addressing hegemonic norms about masculinities (Chu et al., 2005, Levant et al., 2016). In addition, acknowledging the need to expand the gender lens to include perspectives on femininities, Tolman and Porche proposed a measure of ideologies of femininities in early adolescents as a driving force guiding girls’ sexuality development (Tolman & Porche, 2000). Whether such measures function in other cultural settings and more broadly, whether common forms of patriarchy exist in early adolescence across cultures is an empirical question which remains to be explored.

Building on a mixed method approach, the present study seeks to address the following empirical question “Are there common gender expectations about romantic relationships between boys and girls in adolescence that can be identified and measured across cultures?”. We subsequently develop, test and validate cross cultural scales assessing young people’s perceptions of such norms. We focus our attention on gender norms about romantic relationships in early adolescence as sexual development, including sexual attitudes, intimate relations and sexual practices is a primary interest of the Global Early Adolescent Study.

## Methods

### Global early adolescent study

The study draws on the Global Early Adolescent study (GEAS), which is the first study to focus on gender norms in early adolescents and its relation to adolescent health in disadvantaged urban environments globally. The GEAS was implemented through a collaboration of university and research institutions from 15 cities (Table 1): Assiut (Egypt), Baltimore (USA), Blantyre (Malawi), Cape Town (South Africa), Cochabamba (Bolivia), Cuenca (Ecuador), Edinburgh (Scotland), Ghent (Belgium), Hanoi (Vietnam), Ile Ife (Nigeria), Kinshasa (DRC), Nairobi (Kenya), New Delhi (India), Ouagadougou (Burkina Faso), and Shanghai (China). The sites were selected because of ongoing research partnerships across several of these institutions and because they represented distinctly different cultures, social, linguistic and ethnic contexts. Each also has a strong history of research on adolescents in urban poor populations. Table 1 provides a description of the study samples per sub-study and per site.

**Table 1:** Description of Study Populations per site and substudy.

			Qualitative study	Pilot 1 quantitaive study (*N* = 1944) (*N*, %)	Pilot 2 quantitaive study (*N* = 434) (*N*, %)
City	Assiut, Egypt		37	121 (6.2)	77 (17.7)
	Baltimore, USA		33	49 (2.5)	
	Blantyre, Malawi			127 (6.5)	75 (17.3)
	Cuenca, Ecuador			112 (5.8)	52 (12.0)
	Cochabamba, Bolivia			121 (6.2)	
	Edinburgh, UK		32	34 (1.8)	
	Ghent, Belgium		30	123 (6.3)	85 (19.6)
	Hanoi, Vietnam			126 (6.5)	68 (15.7)
	Ile Ife, Nigeria		38	122 (6.3)	
	Kinshasa, DRC			123 (6.3)	77 (17.7)
	Nairobi, Kenya		30	444 (22.8)	
	New Delhi, India			122 (6.3)	
	Ouagadougou, Burkina Faso			124 (6.4)	
	Shanghai, China		34	196 (10.1)	
				
Sex	Boy			944 (48.6)	215 (49.5)
	Girl			997 (51.3)	219 (50.5)
				
Age	10			280 (14.4)	84 (19.4)
	11			417 (21.5)	106 (24.4)
	12			449 (23.1)	85 (19.6)
	13			441 (22.7)	89 (20.5)
	14			354 (18.2)	70 (16.1)
			
% Enrolled in school				1893 (97.4)	424 (97.7)

The present study is based on the formative phase of the GEAS, which took place between June 2014 and April 2017. This formative stage used a mixed method approach to explore gendered transitions into adolescence and to develop a set of cross-cultural instruments for assessing different dimensions of gender norms and health concerns among early adolescents ages 10 to 14 years. While the GEAS study examines a range of gender domains, including perceptions of stereotypical traits (“male toughness” versus “female vulnerability), and perceptions of gender stereotypical roles (male as provider and decision maker versus female as caregiver and subordinate), this paper focuses on the development of cross cultural scales specifically assessing gender norms about romantic relationships between boys and girls in early adolescence. As previously indicated, this focus is informed by the study’s interest in exploring sexual development in early adolescence. We describe the approach that was used to develop the cross-cultural instruments and assess the psychometric properties of the scales.

### Process for developing the gender norms about relationships scales

**Fig. 1:**
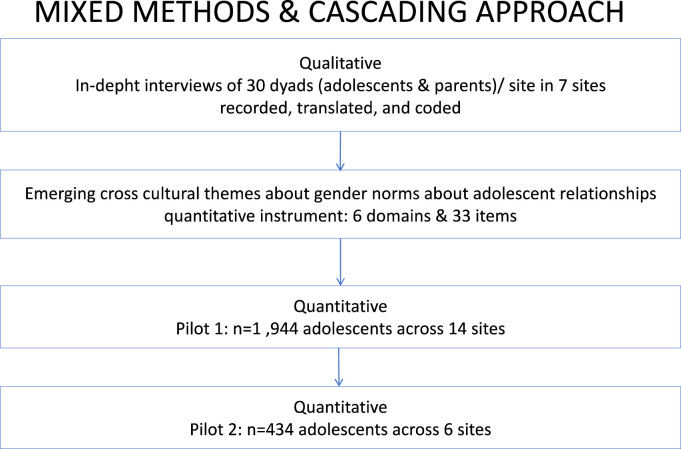
Mixed method approach for scale development.

Unlike previous scales that were developed in one context and adapted to others (e.g. the GEM Scale), the present study followed a bottom up approach, grounding the development of the scales across a diversity of cultures. Specifically, we used a mixed method cascading process, starting with in-depth interviews among adolescents in seven sites to identify the common constructs for a multi-dimensional scale. We then tested the resulting scale for face validity in local languages and piloted it in 14 GEAS sites (Fig. 1). After initial exploratory analysis, a revised set of subscales were tested for validation in six GEAS sites, chosen to reflect the diversity of settings (Table 1). A more detailed description of the qualitative and the quantitative methods used to develop and validate the scales are provided below.

To limit method-induced variation, we used identical protocols for training, study design, data collection and analysis plan across all sites (see next section). The World Health Organization Ethical Review Board, the Johns Hopkins Bloomberg School of Public Health IRB, and each site’s human subject ethics review committee approved all research protocols (qualitative and quantitative).

#### Scale item development

##### Qualitative data collection and procedures

The first step of scale development involved in-depth interviews conducted by trained interviewers, with approximately 30 adolescents per site (15 boys and 15 girls, aged 11 to 13 years), between 2014 and 2015. The interviews aimed at exploring gendered transitions from childhood to adolescents. For this study, we used data from 200 adolescents across seven sites, which included Assiut, Baltimore, Edinburgh, Ghent, Ile Ife, Shanghai, Nairobi. Adolescents at each site, were purposively sampled according to age, sex and in some sites, according to migrant status or household income. We used various sampling strategies, including household selection (Ile-Ife, Nairobi), youth clubs and community-based organizations (Ghent and Baltimore) or religious institutions (Baltimore), or schools (Ghent, Edinburgh, Shanghai, Assiut) (Table 1). All adolescents gave assent and their parents gave consent for their participation. Interviews were conducted in a confidential space and audio-taped. All recordings were transcribed verbatim, translated into English, and uploaded in Atlas.ti. Each local PI proceeded with random checks of translated interviewers. Details of the study procedures are published elsewhere (Mmari et al., 2017).

##### Qualitative data analysis

All quotations that related to gender norms were extracted from the transcripts across sites and uploaded into Dedoose, a qualitative analysis software program. Using this online platform, each country partner coded their own data line by line, contributing to the co-construction of a common set of gender norms codes across sites. This cross-site, finely grained coding resulted in the emergence of six cross-cultural themes related to norms regulating romantic interactions between boys and girls in early adolescence: “boys’ natural attraction to girls”, “boys’ social gains for having girlfriends”, “boys’ normative romantic relationships with girls”, “girls’ social risk for having boyfriends”, “girls’ responsibility to avoid boy interest”, and, “girls’ normative romantic relationships with boys”. These domains constituted the backbone of the multidimensional gender norms instrument and served to populate a bank of items reflecting these domains (Annex A).

##### Administration of scale items & initial assessment of scale properties

The quantitative gender norms instrument contained descriptive and subjunctive norm items all designed on a 5-point Likert Scale (1: Disagree a lot, 2: Disagree a little, 3: Neither agree nor disagree, 4: Agree a little, 5: Agree a lot). The instrument was tested for face validity among 15 adolescents, aged 10–14 years, in each of the 14 sites (Assiut, Baltimore, Blantyre, Cuenca, Cochabamba, Ghent, Edinburg, Hanoi, Ile Ife, Kinshasa, Nairobi, New Delhi, Ouagadougou, Shanghai). It was subsequently piloted among approximately 120 adolescents 10–14 years in each site (and over 400 in Kenya to allow site specific analysis) between November 2015 and September 2016. Adolescents were recruited from households, community youth centers or schools and were invited to complete a survey, which included the gender norms instrument. For all adolescents enrolled in the pilot, assent and parental permission was obtained by the researchers in each site. Depending on level of literacy and technical capacity, the interviews were conducted face to face or were self-administered on mobile tablets using Computer Assisted Self Interview (CASI) or Audio-CASI features (see Table 1 for details about study population). All surveys were uploaded into a secure server and merged into a single file to allow for cross-site analysis.

The same procedures were used in the second pilot conducted between January 2017 and April 2017 among approximately 75 adolescents in each of six sites (Assiut, Blantyre, Cuenca, Ghent, Hanoi, Kinshasa). These sites were chosen to reflect the cultural and geographical diversity of the GEAS (Table 1).

##### Quantitative data analysis

We initially used Pilot 1 survey data from 1944 adolescents for cross-site scale development and subsequent reliability testing. To ensure data quality, we assessed missing patterns of items per respondent and dropped 65 observations for which four or more items were missing. For the remaining sporadic missing items, we used imputation techniques; including K-Nearest Neighbor (kNN with k-value of 35, corresponding to the square root of complete cases). Psychometric criteria (Eigenvalues, Scree test (Cattell, 1966), and Parallel Analysis (Hayton, Allen, & Scarpello, 2004)) were used to determine the number of factors to retain in the final multidimensional scale. Specifically, we conducted principal component analysis, examined scree plots and used parallel analysis to determine the number of initial factors. We ran exploratory factor analysis to exclude items that were not loading on any factor (less than 0.40) or loading mostly on the second factor in case of a two factor solution and reran the analysis on the reduced set of items. We applied orthogonal rotation during Exploratory Factor Analysis (EFA) to retrieve factor loadings because the correlation between factors was not significant (< 0.3). Factor analysis also guided the selection of a more parsimonious set of items loading on the identified factors (based on 0.40 factor loading criteria) (Ford, MacCallum, & Tait, 1986). Each sub-scale was named based on interpretation of items. We computed polychoric ordinal Cronbach’s alphas to assess the internal reliability of each factor. Analyses on complete cases (*N* = 1301) yielded similar results.

##### Refinement and validation of the scales

We validated our previous findings among 434 adolescents using Pilot 2 data, which tested a slightly revised set of Pilot 1 items, informed by Pilot 1 results. Similar to Pilot 1, we performed analyses on both imputed data and complete cases across all six sites. We dropped 15 observations with 30% missing items or higher. In addition to cross-site EFA, we explored factor loading of each subscale within each site guided by cross-site results using kNN imputed data. We computed polychoric ordinal Cronbach alphas for both cross-site and site-specific subscales to assess the internal reliability of the finalized subscales. We checked that analyses conducted on complete cases yielded similar results. Based on the final structure of the gender norms scales, we conducted a descriptive analysis of the gender scale scores by age and sex in each site. Scores were computed as a mean score across items; with each individual item score ranging from 1 to 5, based on the 5 Likert scale response options. Observations with more than 50% items missing on a subscale were dropped from analysis. Mean scores were compared by Student t-test, after examining normal distribution and homoscedasticity. A two-sided p-value of less than 0.05 was considered for statistical significance. STATA Version 14.2, StataCorp LLC, TX and R Version 3.3.3 (R Project) were used for all statistical analysis.

## Results

### Qualitative findings: emergent scale domains

The cross-cultural qualitative analysis produced a total of six domains that related to perceptions of gender norms regulating relationships between boys and girls:

1. “boys’ natural attraction to girls”,
2. “boys’ social gains for having girlfriends”,
3. “boys’ normative romantic relationships with girls”,
4. “girls risk for having boyfriends”,
5. “girls’ responsibility to avoid boy interest”, and
6. “girls’ normative romantic relationships with boys” (Annex A).

Boys and girls alike acknowledged heterosexual romantic involvement in early adolescence, although they were more likely to report their friend’s experiences rather than their own. Such relations followed gendered scripts, portraying boys as naturally attracted to girls and as the natural “initiator” of romantic encounters. By contrast, girls were expected to keep boys at a distance to avoid the negative consequences of sexual relations. Such unequal standards are reflected in this description of a father’s reaction to a son or daughter’s romantic involvement:

I: *Okay, and your daddy? What would he do if he’d know (about boyfriend)?*

R. *Ho, ho, ho… Haha. My farther would certainly say “Nooooo, nooo”, I think*

I: *And why would he say that, you think?*

R: *I don’t know, I don’t know that either. My daddy is rather strict, he appears to be a strict person*

I: *Yeees… And what if your brother would come home and say that he’s in love with someone, what would he say then?*

R: *Hoho, haha. My father tells my brother this: “Come on! Still not in love?” Then my brother starts to laugh. But yeah, if my brother would have someone, then he wouldn’t say anything, you see* (Adolescent girl, Ghent)

Male romantic interest, encouraged by peers, was mostly viewed as a sign of social status of “becoming a man” rather than an emotional commitment. Many boys sought peer recognition by engaging in relationships to “show off” to their friends. This lack of male “authentic” attachment reinforced community perceptions of girls’ vulnerability when engaging in romantic relations as boys are “not to be trusted”. Girls across sites were strongly advised to “stay away from boys” and “cover up” to avoid masculine attention and prevent sexual risks. Internalizing parental advice, young girls saw themselves as responsible for their own safety, but with little ability to control boys’ behavior.

*R: Going with a woman, probably like going with them just for the fun of it, not probably because they like them, just so everybody can see that they are different, like they are the wrong people to mess with.*

*I: Who specifically is he trying to prove something to?*

*R: His friends to show that he is better than the rest of them* (Adolescent girl, Baltimore)

*R: Let us say you get involved with a boy and he keeps giving you presents/gifts and you take. After a while he will demand that you return his things back and if you are not able to pay them back he will demand you exchange that with sex. After that he will dump you and move on and probably you are pregnant. And usually when girls are pregnant they can either abort or their parents refuse to take them in and they end up committing suicide.* (Adolescent girl, Nairobi)

The gendered scripts regarding romantic relations created social hierarchies of power between boys and girls, which favored boys.

*R: (Boys) are the ones who are supposed to look for girls but girls can’t look for boys. (*Adolescent *boy, Nairobi)*

These qualitative findings led to the construction of a multidimensional scale exploring perceptions of gender norms about relationships, distinguishing the six aforementioned dimensions. The qualitative data also served to populate a bank of items reflecting the domains. Items included both descriptive norms “Boys have girlfriends for fun more than love” and injunctive norms “girls should keep boys at a distance”. The bank of items was revised through multiple iterations with country partners, to check for content adequacy across sites, and allow for country specific indicators to be added. Items were translated and back translated in local languages. The resulting multidimensional scale included 33 items covering 6 subdomains (Annex A).

### Quantitative findings: results from exploratory factor analysis

Results from Pilot 1 study suggested a two-factor structure. Two domains, “boys’ natural attraction to girls” and “girls’ responsibility to avoid boy interest” (comprising 8 items), were not consistently captured in the two-factor structure and were removed from the analysis. In addition, 8 items with loadings of less than 0.40 on both factors, were removed subsequently. We included 10 items and 11 items for the first and second factor, respectively. Based on the descriptions of items included for each factor, we labeled the first factor, “adolescent romantic expectations”, reflecting perceptions that boys’ and girls’ romantic interests are normative in early adolescence. The second factor, defined as the “Sexual Double Standard” subscale captures differential gender standards regarding romantic engagement, encouraged for boys who gain social status but devalued for girls who risk their social reputation (Table 2). The Eigenvalue was 5.0 for factor 1 and 4.5 for factor 2. Together, they explained 45% of the total variance. For the “adolescent romantic expectation” subscale, rotated factor loadings ranged from 0.48 to 0.76 with a Polyhoric Cronbach Alpha value of 0.87. For the sexual double standard subscale, rotated factor loadings ranged from 0.47 to 0.69 with a Polyhoric Cronbach’s Alpha of 0.85.

**Table 2:** Rotated Factor Loadings for Gender Norm - Relationship Items: across-sites results from kNN-imputed pilot 1 data (*N* = 1879).

*Item Description*	**Adolescent Romantic Expectations**	**Sexual Double Standard**	**Uniqueness**
It’s ok for a girl your age to be in a relationship with a boy as more than just friends	**0.76**	–0.10	0.41
It’s ok for a boy your age to be in a relationship with a girl as more than just friends	**0.74**	–0.05	0.44
It’s ok for a boy your age to talk and spend time with a girl alone	**0.71**	–0.13	0.48
It’s normal for a girl to want a boyfriend at your age	**0.67**	0.00	0.55
It’s ok for a boy and girl your age to talk and spend time together alone	**0.67**	–0.11	0.53
It’s ok for a boy to have more than one girlfriend at a time	**0.58**	0.19	0.63
It’s ok for a boy to have a lot of girlfriends	**0.57**	0.11	0.66
A girl can have a boyfriend as long as she continues working well in school	**0.57**	0.07	0.67
Boys should have girlfriends to discover love	**0.56**	0.16	0.66
It’s ok for a girl to have more than one boyfriend at the same time	**0.48**	0.16	0.75
Boys fool girls into having sex	-0.17	**0.69**	0.50
Boys have girlfriends to show off to their friends	0.16	**0.67**	0.52
Girls often get into “trouble” when they have boyfriends	-0.27	**0.60**	0.57
Boys lose interest in a girl after they have sex with her	-0.03	**0.59**	0.65
Boys tell girls they love them when they don’t	0.05	**0.58**	0.66
Boys have girlfriends for fun more than love	0.16	**0.58**	0.64
Boys feel they should have girlfriends because their friends do	0.27	**0.58**	0.60
Girls who have boyfriends are irresponsible	–0.23	**0.56**	0.63
Girls are the victims of rumors if they have boyfriends	–0.04	**0.54**	0.70
A girl will lose interest in studying if she has a boyfriend	–0.29	**0.54**	0.63
Girls who have boyfriends are popular	0.20	**0.47**	0.74
*Polychoric Ordinal Cronbach’s Alpha*	*0.87*	*0.85*	
*Eigenvalue*	*5.03*	*4.52*	
*% Total Variance Explained*	*45%*		

After a further round of revisions from country partners, we included 18 items assessing perceptions of gender norms about relationships in Pilot 2. Results from kNN-imputed data (*N* = 419) suggested the same two-factor structure as Pilot 1. We retained 10 items for the sexual double standard subscale and six items for the adolescent romantic expectations (Table 3). The Eigenvalues were 4.8 and 3.1 respectively and the two-factor structure explained 56% of the total variance. Compared with Pilot 1, the rotated factor loadings were improved in pilot 2, ranging from 0.57 to 0.78 for the sexual double standard scale and 0.62 to 0.76 for the adolescent romantic expectations scale. The polychoric ordinal Cronbach alpha value was increased to 0.90 for the sexual double standard and remained stable for the adolescent romantic expectations subscale (0.86).

**Table 3:** Rotated Factor Loadings for Gender Norm - Relationship Items: across-sites results from kNN-imputed pilot 2 data (*N* = 419).

*Item Description*	Sexual Double Standard	Adolescent Romantic Expectations	Uniqueness
Boys have girlfriends to show off to their friends	**0.78**	0.05	0.38
Adolescent boys fool girls into having sex	**0.77**	–0.07	0.40
Boys tell girls they love them when they don’t	**0.74**	0.05	0.45
Adolescent boys lose interest in a girl after they have sex with her	**0.74**	–0.05	0.46
Boys have girlfriends for fun more than love	**0.71**	0.15	0.47
Adolescent girls should avoid boys because they trick them into having sex	**0.66**	–0.21	0.51
Girls who have boyfriends are irresponsible	**0.65**	–0.17	0.55
Girls are the victims of rumors if they have boyfriends	**0.65**	–0.05	0.58
Boys feel they should have girlfriends because their friends do	**0.62**	0.16	0.59
Girls your age often get into “trouble” when they have boyfriends	**0.57**	0.01	0.68
It’s normal for a boy your age to want a girlfriend	–0.06	**0.76**	0.43
A boy should be able to have a girlfriend if he wants to	0.03	**0.75**	0.43
A girl should be able to have a boyfriend if she wants to	–0.01	**0.75**	0.44
It’s normal for a girl to want a boyfriend at your age	–0.12	**0.70**	0.50
Boys should have girlfriends to experience love	0.12	**0.67**	0.53
A boy and a girl your age should be able to spend time together alone if they want to	0.00	**0.62**	0.62
*Ordinal Cronbach’s Alpha*	*0.90*	*0.86*	
*Eigenvalue*	*4.83*	*3.14*	
*Total % of Variance Explained*	*56%*		

To assess the relevance of the two subscales in different cultural settings, we examined the properties of each subscale in each site using Pilot 2 kNN-imputed data. An iterative site-specific analysis resulted in dropping four items from the sexual double standard subscale due to low factor loadings or high level of uniqueness in some sites. The resulting final cross-cultural sexual double standard subscale included six items, with factor loadings ranging from 0.30 to 0.90 and polychoric ordinal Cronbach alpha values all above 0.70 with the exception of Hanoi (0.61) (Table 4). The same process was used to examine the properties of the “adolescent romantic expectations” subscale in each site, which resulted in dropping two items due to low factor loading in some settings (Ghent). The resulting final adolescent romantic expectations subscale included four items (Table 4), with factor loadings ranging from 0.32 to 0.95 and polychoric ordinal Cronbach alpha values above 0.70 in all sites, except Kinshasa (0.64). The results were consistent with site-specific complete cases analysis.

**Table 4:** Rotated Factor Loadings for Sexual Double Standard and Adolescent Romantic Expectations Subscales: site-specific results from kNN-imputed pilot 2 data.

	Assiut (*N* = 75)	Blantyre (*N* = 72)	Cuenca (*N* = 51)	Ghent (*N* = 76)	Hanoi (*N* = 68)	Kinshasa (*N* = 77)
**Sexual double standard**	Factor	Factor	Factor	Factor	Factor	Factor
Boys have girlfriends to show off to their friends	0.58	0.84	0.86	0.76	0.39	0.72
Adolescent boys fool girls into having sex	0.73	0.52	0.90	0.59	0.58	0.72
Boys tell girls they love them when they don’t	0.45	0.72	0.72	0.78	0.60	0.56
Adolescent boys lose interest in a girl after they have sex with her	0.61	0.45	0.79	0.59	0.43	0.71
Adolescent girls should avoid boys because they trick them into having sex	0.63	0.87	0.59	0.43	0.51	0.58
Girls are the victims of rumors if they have boyfriends	0.68	0.30	0.66	0.48	0.40	0.63
*Polychoric Ordinal Cronbach Alpha*	0.80	0.84	0.87	0.73	0.61	0.84
*Total % of Variance Explained*	*48%*	*50%*	*64%*	*48%*	*36%*	*52%*
**Adolescent Romantic Expectations**	Factor	Factor	Factor	Factor	Factor	Factor
It’s normal for a boy your age to want a girlfriend	0.86	0.77	0.80	0.80	0.73	0.95
A boy should be able to have a girlfriend if he wants to	0.81	0.87	0.67	0.71	0.80	0.49
A girl should be able to have a boyfriend if she wants to	0.78	0.92	0.86	0.53	0.75	0.32
It’s normal for a girl to want a boyfriend at your age	0.89	0.72	0.60	0.76	0.71	0.60
*Polychoric Ordinal Cronbach Alpha*	0.90	0.89	0.79	0.72	0.81	0.64
*Total % of Variance Explained*	77%	75%	65%	62%	67%	51%

### Descriptive scale score by site, age and sex

Based on the final versions of each subscale (six and four items respectively), we assessed mean scores by gender and age in each Pilot 2 site (Table 5). In general, girls scored higher than boys on the sexual double standard scale and lower on “adolescent romantic expectations” suggestive of more conservative views about relationships than boys. These gender differences were significant in Assiut and Ghent for the sexual double standard in Assiut and Blantyre and for the “adolescent romantic expectations”. Older adolescents (13–14 years) scored higher on the adolescent romantic expectations scale than their younger peers (10–12 years) in four out of six sites. At the same time, older adolescents were more likely to endorse the sexual double standard, although age differences were not statistically significant in any site.

**Table 5:** Mean score and standard errors per site (based on Pilot 2 non-imputed data).

Sexual Double Standard	Total (*n* = 417)	Assiut (*n* = 75)	Blantyre (*n* = 73)	Cuenca (*n* = 50)	Ghent (*n* = 74)	Hanoi (*n* = 68)	Kinshasa (*n* = 77)
Gender							
Girls	3.6 (0.1)*	4.4 (0.1)**	4.0 (0.1)**	3.4 (0.2)	2.8 (0.2)*	2.6 (0.1)	4.1 (0.1)
Boys	3.4 (0.1)	3.6 (0.2)	4.5 (0.1)	3.5 (0.2)	2.4 (0.1)	2.6 (0.1)	3.7 (0.2)
							
Age							
10–12	3.3 (0.1)**	3.9 (0.1)	4.1 (0.2)	3.4 (0.2)	2.6 (0.1)	2.6 (0.1)	3.8 (0.2)
13–14	3.8 (0.1)	4.1 (0.1)	4.3 (0.1)	3.6 (0.2)	2.9 (0.3)	2.7 (0.1)	4.1 (0.2)

Adolescent Romantic Expectations	Total (*n* = 425)	Assiut (*n* = 75)	Blantyre (*n* = 71)	Cuenca (*n* = 52)	Ghent (*n* = 82)	Hanoi (*n* = 68)	Kinshasa (*n* = 77)

							
Gender							
Girls	2.9 (0.1)**	2.4 (0.2)**	2.4 (0.2)*	3.8 (0.2)	3.3 (0.1)	2.8 (0.2)	2.7 (0.2)
Boys	3.3 (0.1)	3.3 (0.3)	3.1 (0.2)	3.9 (0.2)	3.5 (0.1)	3.1 (0.2)	2.8 (0.2)
							
Age							
10–12	3.0 (0.1)	3.0 (0.2)	2.9 (0.2)	3.6 (0.2)**	3.3 (0.1)**	2.7 (0.2)**	2.5 (0.2)**
13–14	3.2 (0.1)	2.7 (0.2)	2.6 (0.2)	4.2 (0.1)	4.1 (0.3)	3.4 (0.2)	3.1 (0.2)

** p ≤ 0.001.

* p ≤ 0.05.

## Discussion

This study used a mixed-method approach, grounded in the voices of young people around the world, to construct cross-cultural scales to assess gender norms regulating relationships in early adolescence. The resulting scales capture two complimentary dimensions of romantic relationships among early adolescents: one measures the expectations about adolescent boy/girl romantic involvement and the other highlights a sexual double standard that encourages boys to pursue sexual and romantic interests and at the same time that it restrains girls from doing likewise.

Our findings complement current measures of norms about masculinities and femininities developed for early adolescents in the United States (Chu et al., 2005) as well as the adapted GEM scale for young adolescents recently tested in Uganda (Vu et al., 2017), showing that stereotypical views manifest at an early stage of development, when young people come to organize their sense of self in relation to others (Erikson, 1968), particularly with respect to gender and sexuality (Galambos, Almeida, & Petersen, 1990). Our study expands on this work by drawing attention to 1) differential gender scripts informing romantic engagement regardless of sexual involvement, 2) conflicting norms regarding girl’s romantic attachment, considered normative but detrimental; and 3) the resonance of these norms across diverse geographies and cultures.

Our study suggests that the sexual double standard, first described by Reis in the late 1950s as a set of norms holding men and women to different standards with regards to premarital sexual interactions (Reiss, 1956), takes root early in adolescence and echoes across cultures. The sexual double standard, pervasive even as societies evolve towards greater sexual permissiveness (Crawford & Popp, 2003), has profound implications for women’s and girl’s health and well-being, restraining their autonomy while exposing them to greater sexual risks due to lack of knowledge and preparedness (Ahlberg, Jylkäs, & Krantz, 2001). However, until now, this research on the sexual double standard had focused on older adolescents or adults (Crawford & Popp, 2003), while its expression in earlier stages of sexual development remained largely unexplored. In our study, we show that even before young people engage in sexual activity, they internalize different “social rules” about acceptable heterosexual romantic engagement for boys versus girls (Blum et al., 2017). These differentials are likely to inform imbalanced power dynamics between partners that shape sexual relations and hinder preventive practices.

While the sexual double standard captures differential gender standards for boys and girls to engage in romantic relationships, the adolescent romantic expectations scale measures the extent to which adolescent girls and boys perceive these relationships to be a normal part of their lives. As such, this cross-cultural measure contributes to the understanding of the ways social expectations surrounding adolescent relationships vary across cultures (as suggested in the differences in mean scores by site observed in our study). Combined with the sexual double standard, the adolescent romantic expectations scale is an opportunity to assess how girls and boys navigate conflicting and mutually reinforcing norms of romantic relationships and the extent to which they both influence actual romantic experiences and overall adolescent development.

This study on gender norms about relationships started as an empirical question “Are there common gender expectations for boys and girls regarding romantic relationships in adolescence that can be identified and measured across cultures”? We partly address this question by showing that measures of gendered romantic relationship expectations and of the sexual double standard are relevant in very diverse cultural settings. Such cross cultural measures are important in monitoring and comparing gender scripts across time and space, to inform global progress towards achieving the United Nations’ 5^th^ Sustainable Development Goal on gender equality by 2030 (United Nations Economic Council, 2017). Likewise, these measures could be used to evaluate the impact of interventions addressing harmful gender norms and to test theories of change linking gender norms to adolescent sexual health and wellbeing. In fact, as part of our second phase of the Global Early Adolescent Study, we are using the scales to test the impact of interventions addressing gender attitudes in five sites across three continents.

While our study proposes a comprehensive approach, using qualitative and quantitative methods to empirically test the existence of common gender scripts, it is not without limitations. We rely on convenience samples of urban poor adolescents. They are not necessarily representative of their communities, and do not capture the diversity of the global population of early adolescents from different social, cultural backgrounds and geographies. Our findings are nevertheless consistent with the Vu study in Uganda, indicating that boys hold more equitable attitudes about relationships than girls in early adolescents (Vu et al., 2017). Further analysis is warranted to understand gender differences in perceptions of adolescent romantic expectations, to assess if girls’ more tradition views are based on experiences of unequal relationships or informed by gender differences in socialization processes, involving family and peers in early adolescence.

While the GEAS includes questions on perceptions of same sex relations, these questions were not included in all sites due to the sensitivity and legality of same sex relations in some locations and the lack of variation in young people’s views about these relationships in other sites. Preliminary analysis also indicated that perceptions of same sex relations did not scale on the same factors as perceptions of heterosexual relations. Subsequent analysis of normative views about same sex relations in early adolescent will be conducted in GEAS sites that have included these questions.

The qualitative analysis suggested a number of common themes but their operationalization into quantitative instruments proved challenging. The six qualitative themes translated in two reconfigured quantitative measures that scaled across cultures, the sexual double standard capturing “boys’ social gains for having girlfriends” and “girls risk for having boyfriends” and the adolescent romantic expectations including “boys’ normative romantic relationships with girls” and “girls’ normative romantic relationships with boys”. We were unable to find a cross-site solution for the two following themes “boys’ natural attraction to girls” and “girls’ responsibility to avoid boys’ interest”, which had fewer items. Further investigation of these domains would require additional items to improve the psychometric properties of these constructs. Moreover, site-specific analysis resulted in the exclusion of a number of items that did not consistently work across sites. This item selection process stresses the limits of cross-cultural measures, that delineate the contours of what is generalizable to the expense of the culturally-specific. The trade-off is necessary for cross-cultural comparisons, but should be reconsidered for site-specific analysis, by enriching the measures with locally grounded expressions about the sexual double standard and adolescent romantic expectations among early adolescents. While both pilot surveys included overall large samples, the site-specific analysis was restricted to much smaller samples, limiting both the statistical analysis and generalizability of the results. The subsequent longitudinal phase of the GEAS, including much larger cohorts of adolescents in specific sites will provide an opportunity to replicate this analysis and explore the correlates of gender norms and the linkages between gender norms and adolescent health indicators. This next step is critical in informing strategies that situate gender at the heart of programs promoting adolescent sexual health.

## Conclusion

Grounded in the voice of young people across five continents, this study revealed common gendered scripts about romantic relationships in early adolescence that led to the development of cross cultural scales. The scales capture two dimensions of gender norms about heterosexual romantic relationships, one that measures adolescent romantic expectations and the other that highlights a sexual double standard that favors boys over girls in romantic and sexual relationships. The findings illustrate that social hierarchies of power in romantic relationships form early in adolescence, regardless of the cultural setting.

## Declarations of interest

None.

## Figures and Tables

**Table A1:** Description of Subdomains and Items Included in Pilot 1.

Subdomain	Item description
Boys’ have girlfriends for social gain rather than love	Boys have girlfriends to show off to their friends
	Boys should have girlfriends to discover love
	Boys feel they should have girlfriends because their friends do
	Boys have girlfriends for fun more than love
	Boys tell girls they love them when they don’t
	Boys lose interest in a girl after they have sex with her
	Boys fool girls into having sex
	
Boys are naturally attracted to girls	Boys are supposed to be attracted to girls
	It’s in boys’ nature to chase girls
	Boys generally compete for the prettiest girls
	Boys like girls who wear revealing clothes
	
Girls’ responsibility to avoid boy interest	Girls should cover up or they will attract unwanted sexual attention
	Girls should be careful about the way they look so they are not seen as trying to seduce men
	It’s a girl’s fault if boys come onto them
	Girls wear short dresses to get boys’ attention
	
Girls should keep boys at a distance or they will get in trouble	Girls should keep boys at a distance
	It’s not good for a girl to spend time alone with a boy
	Girls often get into “trouble” when they have boyfriends
	Girls who have boyfriends are irresponsible
	Girls should be aware that boys can take advantage of them
	Girls are the victims of rumors if they have boyfriends
	A girl will lose interest in studying if she has a boyfriend
	
Hetero-normative relationships for boys	It’s ok for a boy your age to be in a relationship with a girl as more than just friends
	Boys judge girls on their character rather than on their looks
	It’s ok for a boy to have a lot of girlfriends
	It’s ok for a boy to have more than one girlfriend at a time
	It’s ok for a boy and girl your age to talk and spend time together alone
	It’s ok for a boy your age to talk and spend time with a girl alone
	
Hetero-normative relationships for girls	It’s ok for a girl to have more than one boyfriend at the same time
	It’s normal for a girl to want a boyfriend at your age
	Girls who have boyfriends are popular
	A girl can have a boyfriend as long as she continues working well in school
	It’s ok for a girl your age to be in a relationship with a boy as more than just friends
